# Genetic Variation in *CYP17A1* Is Associated with Arterial Stiffness in Diabetic Subjects

**DOI:** 10.1155/2012/827172

**Published:** 2012-10-23

**Authors:** Soo Jin Yang, Seung-Tae Lee, Won Jun Kim, Se Eun Park, Sung Woo Park, Jong-Won Kim, Cheol-Young Park

**Affiliations:** ^1^Department of Food and Nutrition, Chonnam National University, Gwangju 500-757, Republic of Korea; ^2^Human Ecology Research Institute, Chonnam National University, Gwangju 500-757, Republic of Korea; ^3^Department of Laboratory Medicine and Genetics, Samsung Medical Center, Sungkyunkwan University School of Medicine, Seoul 135-710, Republic of Korea; ^4^Center for Genome Research, Samsung Biomedical Research Institute, Samsung Medical Center, Seoul 135-710, Republic of Korea; ^5^Division of Endocrinology and Metabolism, Department of Internal Medicine, Gangneung Asan Hospital, University of Ulsan College of Medicine, Gangneung 210-711, Republic of Korea; ^6^Division of Endocrinology and Metabolism, Department of Internal Medicine, Kangbuk Samsung Hospital, Sungkyunkwan University School of Medicine, Seoul 110-746, Republic of Korea

## Abstract

Hypertension and arterial stiffness are associated with an increasing risk of diabetes and cardiovascular diseases. This study aimed to identify genetic variants affecting hypertension and arterial stiffness in diabetic subjects and to compare genetic associations with hypertension between prediabetic and diabetic subjects. A total of 1,069 participants (326 prediabetic and 743 diabetic subjects) were assessed to determine the genetic variants affecting hypertension by analyzing 52 SNPs previously reported to be associated with hypertension. Moreover, the SNPs were tested for association with hemodynamic parameters related to hypertension. Out of the 52 SNPs analyzed, four SNPs including rs5326 (*DRD1*), rs1004467 (*CYP17A1*), rs2960306 (*GRK4*), and rs11191548 (near *NT5C2*) in diabetic subjects and rs1530440 (*C10orf107*) in prediabetic subjects showed a modest association with hypertension (*P* = 0.0265, 0.0020, 0.0066, 0.0078, and 0.0015, resp; all were insignificant after Bonferroni correction). Of these SNPs, rs1004467 in *CYP17A1* was significantly associated with augmentation index in diabetic subjects who were not taking antihypertensive medication (*P* = 0.0001; corrected *P* = 0.006) but not in diabetic subjects receiving antihypertensive medication. This finding suggests that certain genetic variations found in diabetic subjects may confer arterial stiffness and the development of hypertension and also be affected by antihypertensive medication.

## 1. Introduction

Hypertension is a major health concern that is increasing worldwide [1] and is associated with an increasing risk of developing diabetes and kidney and cardiovascular diseases [2, 3]. The etiology of hypertension is complex in that both genetic and environmental factors influence its development. So far, enormous efforts have been made to identify common genetic variants affecting hypertension by conducting several large-scale genomewide association (GWA) studies including the Wellcome Trust Case Control Consortium (WTCCC) study and the Framingham Heart Study 100K Project [4–7]. However, to our knowledge, no studies have reported a genetic association with hypertension relating to diabetes. Given that numerous genes are known to be associated with hypertension and the prevalence of hypertension in diabetic subjects is relatively high, it is likely that allele variations for multiple genes previously reported to be associated with hypertension may also influence the development of hypertension in the diabetic population. In addition, it is possible that there will be different patterns of association with developing hypertension in prediabetic versus diabetic subjects.

Arterial stiffness, characterized by hardening and decreased elasticity of the arteries, is considered as a marker of vascular aging and a cardiovascular risk factor [8]. It can be assessed noninvasively by established standard measures including pulse wave velocity (PWV) and augmentation index (AI) [9]. Arterial stiffness is associated with hypertension, diabetes, obesity, cardiovascular diseases, and aging [9–11]. In addition, increased arterial stiffness and hypertension aggravate each other through a vicious cycle [12]. It is thought that diabetes and genetic variations associated with the development of hypertension may contribute to the vicious cycle of aggravation between arterial stiffness and hypertension.

Here, we aimed to identify genetic variants affecting the development of hypertension and arterial stiffness in diabetic subjects and to compare associations with hypertension between prediabetic and diabetic subjects by analyzing 52 single nucleotide polymorphisms (SNPs) previously reported to be associated with hypertension. In addition, the SNPs were tested for associations with hemodynamic parameters including measures of peripheral and central blood pressure, pulse pressure (PP), PWV, and AI. Our findings demonstrate that there was a different association pattern with hypertension between prediabetic and diabetic subjects. Moreover, rs1004467 in the cytochrome P450, family 17, subfamily A, polypeptide 1 *(CYP17A1*) gene was significantly associated with AI in diabetic subjects without antihypertensive medication.

## 2. Subjects and Methods

### 2.1. Subjects

The study subjects were selected from the Seoul Metro-City Diabetes Prevention Program (SMC-DPP), which is a community-based 5-year follow-up program composed of pre-diabetes and diabetes arms with enrollment from 6 public health centers in Seoul, Korea. From August 2009 to December 2009, a total of 1,069 volunteers between the ages of 20 to 65 years (mean age 53 ± 0 years) were selected for the present analysis; 621 participants were men (58%), and 364 participants had hypertension (34%). Subjects consisted of 326 prediabetic and 743 diabetic individuals. Participants were excluded from the study if they were younger than 20 years or older than 65 years or had type 1 diabetes or history of malignancy. The institutional review board of Sungkyunkwan University Kangbuk Samsung Hospital approved the study protocol, and a written informed consent was obtained from all participants. 

### 2.2. Clinical Measurements

Trained nurses administered a questionnaire to collect information about the participants' medications, history of hypertension, and anthropometric parameters. Peripheral and central blood pressures were measured in a quiet room following a 10 min rest period in the supine position. Peripheral blood pressures were measured with a mercury sphygmomanometer. Phases I and V of Korotkoff sounds were considered as systolic blood pressure (SBP) and diastolic blood pressure (DBP), respectively. Hypertension was diagnosed as SBP ≥140 mmHg, DBP ≥90 mmHg, a current use of antihypertensive medications, or history of hypertension. Central blood pressures were estimated by applanation tonometry of the radial artery at the left wrist (Omron HEM-9000AI; Omron Healthcare, Kyoto, Japan). All blood samples were drawn after an overnight fast at the time of admission. Glucose, hemoglobin A_1c_ (HbA_1c_), insulin, C-peptide, and high-sensitivity C-reactive protein (hs CRP) were measured by standard laboratory methods. Pre-diabetes was defined as subjects with fasting plasma glucose of 5.6–6.9 mmol/L and/or 2 h glucose concentration of 7.8–11.0 mmol/L after a 75 g oral glucose load. Diabetes was defined as subjects with a history of known diabetes, or with fasting plasma glucose of >7.0 mmol/L and/or 2 h glucose concentration of >11.1 mmol/L after a 75 g oral glucose load. Triglyceride (TG), total cholesterol, and high-density lipoprotein cholesterol and low-density lipoprotein cholesterol levels were measured by an enzymatic colorimetric assay (ADVIA 1800; Siemens, Deerfield, IL, USA). 

### 2.3. Arterial Stiffness

Arterial stiffness was assessed by measuring PWV and AI using an automatic waveform analyzer (VP-2000; Colin, Komaki, Japan). Pressure waveforms of the brachial and tibial arteries were recorded by an oscillometric method using the occlusion/sensing cuffs adapted to both arms and both ankles. Pressure waveforms of the carotid and femoral arteries were recorded using multielement tonometry sensors placed at the left carotid and the left femoral arteries. Electrocardiogram was monitored with electrodes placed on both wrists. The first two heart sounds, S1 and S2, were detected by a microphone set placed on the left edge of the sternum at the third intercostal space. The waveform analyzer measures time intervals between S2 and the notch of the carotid pulse wave (Thc), between pulse waves of the carotid and brachial arteries (Tcb), and between S2 and the notch of the femoral arteries (Thf). Also, the waveform analyzer estimates the path lengths of the heart-carotid (Dhc), the carotid-brachial (Dcb), and the heart-femoral (Dhf) segments on the basis of height. PWV was calculated for each arterial segment as the path length divided by the corresponding time interval. Augmented pressure was determined as the pressure difference between the first and second peaks of carotid waveform. AI was calculated as augmented pressure/central PP. To minimize the influence of acute smoking on measurements, all subjects were asked to abstain from smoking at least 12 hours before measurement.

### 2.4. Selection of SNPs and Genotyping

Candidate SNPs were selected from recent GWA studies and large candidate gene association studies [4, 6, 7, 13–20]. Whole blood specimens were collected from each individual into EDTA tubes, and genomic DNA was isolated from peripheral blood leukocytes using the Wizard Genomic DNA Purification Kit according to the manufacturer's instructions (Promega, Madison, WI, USA). Multiplex SNP genotyping was performed using primer extension and the matrix-assisted laser desorption/ionization time-of-flight (MALDI-TOF) mass spectrometry using iPLEX Gold technology from Sequenom (Sequenom, San Diego, CA, USA). Primer design, PCR, and spectra analysis were done according to the standard iPLEX methodology. Quality control was performed by excluding individual SNPs or samples with genotype call rates less than 95% and SNP assays with poor quality spectra/cluster plots. After excluding SNPs with a minor allele frequency (MAF) < 0.05 or Hardy-Weinberg equilibrium *P* < 0.001, total 52 SNPs were analyzed for associations with hypertension.

### 2.5. Statistical Analysis

SAS 9.2 (SAS Inc., Cary, NC, USA) and SNP & Variation Suite (SVS) 7 (Golden Helix Inc., Bozeman, MA, USA) software programs were used to perform all statistical analyses. Data were expressed as mean ± SD. The significance of differences between the groups was evaluated through Student's *t*-test or one-way analysis of variance. The differences in frequencies between groups were tested for statistical significance with *χ* ^2^ tests. The association between genotypes and hypertension was analyzed by multivariate logistic regression analysis after adjustment for age, sex, body mass index, and duration of diabetes. The association between each SNP and hypertension was examined through the use of four different models (minor allele dominant, minor allele recessive, minor allele additive, and MAF models). Among four association analysis models, the results using a minor allele dominant mode were reported because the analysis showed the strongest association. Taking into account that 52 SNPs were tested in parallel, associations between the SNPs and hypertension were assessed after Bonferroni correction for multiple testing. The association between genotypes and measures of hypertension and arterial stiffness was analyzed by multivariate linear and median regression analyses after adjustment for age, sex, body mass index, and duration of diabetes. Values of *P* < 0.05 were considered statistically significant. 

## 3. Results

### 3.1. Clinical Parameters of Study Subjects

Clinical and biochemical parameters of the prediabetic and diabetic subjects are summarized in Table 1. The blood biochemical parameters in diabetic subjects were assessed before diabetic medication, and, as expected, diabetic subjects had higher fasting blood glucose, HbA_1c_, fasting insulin and C-peptide, hs CRP, and TG than prediabetic subjects. The disease duration in diabetic subjects was 5.7 ± 5.8 years, and 561 (75.5%) of the diabetic subjects had received diabetic medication for 5.9 ± 5.3 years. The usage of antidyslipidemia medication and the number of smokers were higher in diabetic subjects compared with prediabetic subjects. Also, diabetic subjects had a higher incidence of concurrent hypertension (43.6%) when compared with prediabetic subjects (12.3%).

We assessed various hemodynamic parameters related to hypertension in subjects with diabetes. In general, hypertensive subjects had higher blood pressures, AI, and PWVs than normotensive ones (Table 2). Since the hemodynamic parameters were measured during medication in subjects taking antihypertensive drugs, we subdivided the subjects according to their medication status. A total of 287 (38.6%) diabetic subjects received antihypertensive medication, whereas none of the 40 hypertensive prediabetic subjects received any medication. Accordingly, the genetic association study was performed on the following three groups: (1) pre-diabetes, (2) diabetes without hypertensive medication, and (3) diabetes with hypertensive medication. 

### 3.2. Genotypes and Their Association with Hypertension

Of the 57 candidate SNPs selected, 52 SNPs had acceptable QC values and MAF greater than 0.05 and thus were eligible for statistical analysis (see Supplementary Table S1 at doi:10.1155/2012/827172). A logistic regression analysis with covariates of age, sex, body mass index, and duration of diabetes showed that four SNPs including rs5326, rs1004467, rs2960306, and rs11191548 were associated with the occurrence of hypertension in diabetic subjects (*P* = 0.0265, 0.0020, 0.0066, and 0.0078, resp.); however, none of these SNPs were significant after Bonferroni correction. In prediabetic subjects, only one SNP, rs1530440, was associated with the occurrence of hypertension (*P* = 0.0015) but was insignificant after Bonferroni correction (Table 3). 

**Figure 1:**
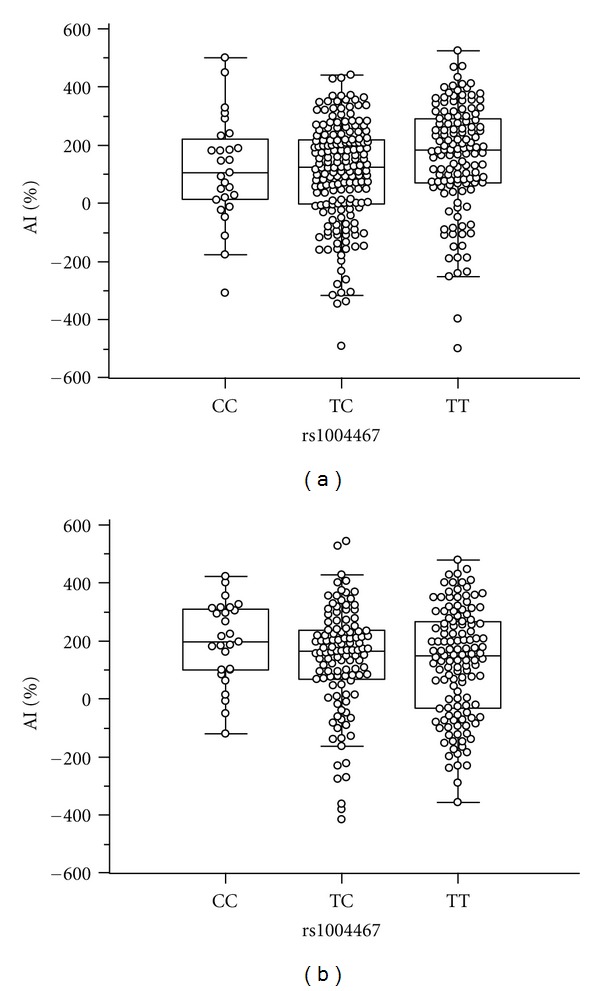
Hemodynamic parameters according to *CYP17A1* rs1004467 genotypes. In diabetic subjects without antihypertensive medication, subjects with TT genotype of rs1004467 tend to have higher AI values than those with CC or TC genotypes (a). This trend was not observed in subjects receiving antihypertensive medication (b).

**Figure 2:**
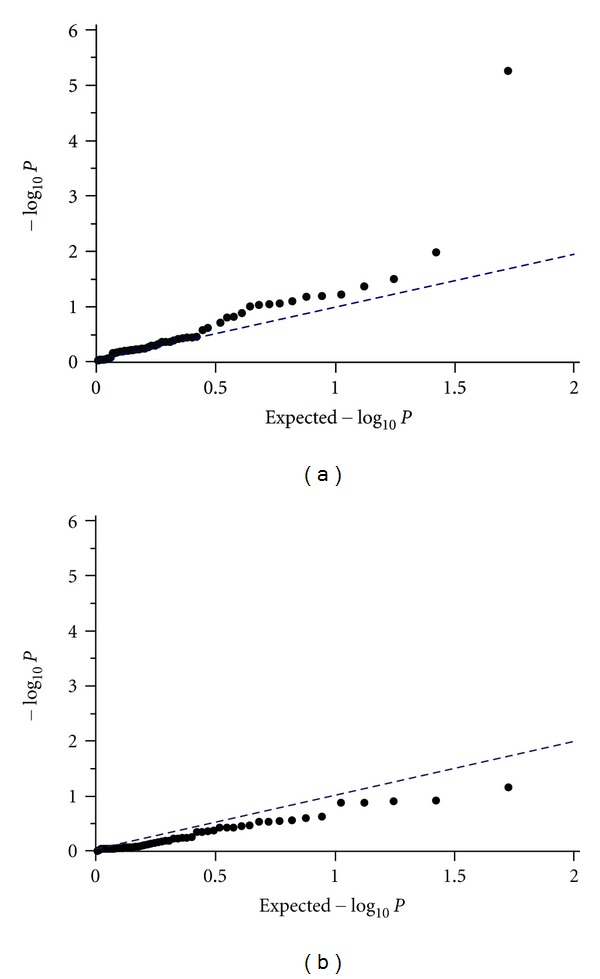
Quantile-quantile plot for expected and observed −log⁡_10_ *P* values. Distributions of *P* values illustrate little evidence of an overall systematic bias. *CYP17A1* rs1004467 showed an excess of low *P* values possibly from true associations in subjects without antihypertensive medication (a). This excess was not evident in subjects receiving antihypertensive medication (b).

To test for associations between the SNPs and hemodynamic parameters, multiple linear or median regression analyses with covariates of age, sex, BMI, and duration of diabetes were performed. Several SNPs showed associations with variable significances; however, only one SNP, rs1004467 in *CYP17A1*, was significant after Bonferroni correction (Table 4). This SNP was strongly associated with AI in diabetic subjects without antihypertensive medication (*P* = 0.0001), even when Bonferroni corrected (*P* = 0.006). In diabetic subjects without antihypertensive medication, AI values tended to be higher in subjects with TT genotype than those with CC, or TC genotypes; AIs were 105.7 ± 185.2, 102.0 ± 180.6 and 162.2 ± 183.1% for CC, TC, and TT genotypes, respectively (Figure 1(a)). The quantile-quantile (Q-Q) plot demonstrates that *P* value distributions across the tested SNPs show little evidence of overall systematic bias, and the excess of low *P* values for rs1004467 is consistent with the presence of true associations (Figure 2(a)). Because most of the diabetic subjects without antihypertensive medication were normotensive, we performed an association analysis in the normotensive diabetic subjects; the results from this analysis showed a significant association (*P* = 0.0001; corrected *P* = 0.005). However, such a trend was not evident in diabetic subjects receiving antihypertensive medication (194.6 ± 139.7, 136.5 ± 178.9, and 123.9 ± 186.9% for CC, TC and TT genotypes, resp. Figures 1(b) and 2(b)). With respect to the other hemodynamic parameters evaluated such as PP and PWVs, no significant associations with the SNPs examined were found. 

## 4. Discussion

Cardiovascular disease is the main cause of death in diabetic subjects and is associated with hypertension and increased arterial stiffness in both general and diabetic populations [3, 21]. The assessment of arterial stiffness allows for a well-established hemodynamic phenomenon in cardiovascular physiology that is negatively correlated with age and positively correlated with heart rate [22]. Higher PWV or AI is known to be associated with endothelial dysfunction and atherosclerosis. Increased arterial stiffness was considered as a marker of subclinical target organ damage and was frequently observed in subjects with high risk for atherosclerosis or patients with developed vascular complications. The development of hypertension and arterial stiffness is complex and affected by various personal environmental and genetic factors. Since genetic transposition is thought to play an important role, efforts have been made to identify genes associated with cardiovascular complications. GWA studies in the general population have found several candidate genes; however, the target phenotypes in these studies were mostly limited to resting blood pressure measured at a single time point due to the large sample sizes and difficulties in phenotypic measurements [23]. Such studies have been very limited in the diabetic population; therefore, we investigated diabetic subjects for GWA hits found in the general population to see if the candidate genes identified from those studies might also have a significant effect in subjects with diabetes. 

The results of our study, however, showed no apparently significant association between previous GWA hits and hypertension and/or blood pressures. The possible reasons for this may include the small number of subjects of the present study and the modest association of the candidate genes. Furthermore, we could not measure the untreated blood pressures of subjects receiving antihypertensive medication, which made it impossible to evaluate the association between genotypes and blood pressures within a high blood pressure range. However, within a normal blood pressure range, we did find a strikingly significant association between *CYP17A1* and AI, one of the most relevant markers for arterial stiffness. The *CYP17A1* gene encodes a key cytochrome P450 enzyme important for the production of sex hormones, mineralocorticoids, and glucocorticoids. Mutations in *CYP17A1* gene typically cause congenital adrenal hyperplasia and hypokalemic hypertension [24]. This gene was shown to be consistently and significantly associated with SBP and DBP in the two large GWA meta-analyses, the Cohorts for Heart and Aging Research in Genome Epidemiology (CHARGE) Consortium [6] and the Global Blood Pressure Genetics (Global BPgen) Consortium [7], and subsequently cross-validated in Korean and Japanese populations [25, 26]. In our diabetic subjects, rs1004467 in *CYP17A1* had the most significant *P* value among the SNPs examined for hypertension; however, the association was only modest (*P* = 0.002; corrected *P* = 0.104). 

Because we found a stronger association between *CYP17A1* and AI in normotensive subjects, we hypothesized that arterial stiffness could be affected more by a certain genotype before the development of hypertension. Indeed, recent studies have shown that increased arterial stiffness can be an early marker of hypertension [27, 28], and salt-induced arterial stiffness can occur in the absence of a change in blood pressure [29]. Another notable finding of our study was the lack of evidence for genotypic associations in subjects receiving antihypertensive medication. As shown in the Q-Q plots, the strong excess of *P* values seen in subjects without antihypertensive medication was not observed in those receiving antihypertensive medication. The AI trends for rs1004467 genotypes were different between subjects with and without medication. One possible theory is that the administration of antihypertensive drugs may alter genotypic effects on arterial stiffness. Many researchers also support that antihypertensive drugs such as angiotensin-converting enzyme inhibitors have an ameliorating effect on arterial stiffness [30–32]. 

For other arterial stiffness markers such as PP and PWVs, *CYP17A1* was not significantly associated and several other genes showed only modest associations. These differences could be attributed in part to the heterogeneity of the arterial tree and the different implications of each marker; for example, aortic PWV more preferentially reflects regional stiffness and AI rather reflects systemic stiffness, although both may largely overlap [33]. In diabetes, early vascular changes mainly affect elastic arteries and muscular arteries and lead to subject-specific hemodynamics [34]. Another consideration is that our study enrolled relatively young subjects and thus may be affected by different age-related properties of the parameters. Recent studies suggest that AI might be a more significant marker of arterial stiffness in younger individuals whereas aortic PWV is likely to be a better marker in elderly individuals [33, 35, 36]. 

There are some limitations in this study. First, this study was cross-sectional and examined the associations between single PWV measures or AI and SNPs. Therefore, we could not exclude the influence of short-term changes in PWVs during the study and it is possible that the timing of the data collection during the study period might have influenced the results. Second, other factors except age, sex, body mass index, and duration of diabetes are known to influence arterial stiffness. We could not analyze the association between genotypes and all clinical parameters; serum creatinine levels were measured only in diabetic arm of study design, and we could not adjust for renal impairment. Also, dyslipidemia, history of chronic vascular diseases, some medications, and smoking status could be confounding factors in this study. Third, we did not screen for secondary hypertensions in the enrollment of subjects.

Despite these limitations, the current study investigated diabetic subjects for candidate SNPs suggested from the general population and demonstrated a significant association of *CYP17A1* with AI in diabetic subjects without antihypertensive medication and a modest association with some of the other candidate genes. The data presented here suggest that certain genetic variations in diabetic subjects may specifically affect arterial stiffness and the development of hypertension and also be affected by antihypertensive medication. 

## Supplementary Material

List of single nucleotide polymorphisms (SNP) investigated in the present study.

## Figures and Tables

**Table 1:** Clinical and biochemical parameters of prediabetic and diabetic subjects.

	Prediabetes	Diabetes	*P* value
*N*	326	743	
Men; *n* (%)	176 (54.0%)	445 (59.9%)	0.069
Age (years)	48 ± 9	55 ± 7	<0.001
Body mass index (BMI; kg/m^2^)	24 ± 3	25 ± 3	0.046
Fasting blood glucose (FBG; mg/dL)	111.7 ± 7.2	151.4 ± 39.6	<0.001
Hemoglobin A_1c_ (HbA_1c_; %)	5.6 ± 0.3	7.3 ± 1.4	<0.001
Fasting insulin (pmol/L)	61.8 ± 25.0	66.0 ± 31.9	0.041
Fasting C-peptide (nmol/L)	0.8 ± 0.4	0.9 ± 0.5	0.001
High-sensitivity CRP (mg/L)	0.13 ± 0.02	0.22 ± 0.02	0.008
Triglyceride (mmol/L)	1.4 ± 0.0	1.8 ± 0.0	<0.001
Total cholesterol (mmol/L)	5.1 ± 0.1	4.9 ± 0.1	0.060
HDL cholesterol (mmol/L)	1.33 ± 0.02	1.27 ± 0.01	0.002
LDL cholesterol (mmol/L)	3.0 ± 0.1	2.9 ± 0.0	0.083
Duration of diabetes (years)	—	5.7 ± 5.8	
Diabetic medication; *n* (%)	—	561 (75.5%)	
Antidyslipidemia medication, *n* (%)	26 (7.9%)	149 (20.1%)	0.001
Smoking status, *n* (%)	41 (12.7%)	164 (22.1%)	0.001
Hypertension; *n* (%)	40 (12.3%)	324 (43.6%)	<0.001

CRP: C-reactive protein; HDL: high-density lipoprotein; LDL: low-density lipoprotein.

**Table 2:** Clinical and hemodynamic parameters of prediabetic and diabetic subjects.

	Prediabetes			Diabetes	
	Normotensive	Hypertensive	Normotensive	Hypertensive	
				Without medication	With medication
*N*	286	40	419	37	287
Men; *n* (%)	152 (53%)	24 (60%)	247 (59%)	26 (70%)	172 (60%)
Age (years)	47.2 ± 8.7^a^	53.6 ± 5.5^b^	53.3 ± 7.6^b^	52.6 ± 8.7^b^	57.0 ± 5.8^c^
Body mass index (BMI; kg/m^2^)	24.3 ± 3.0^a^	25.0 ± 2.8^ab^	24.1 ± 3.2^a^	25.6 ± 3.3^b^	25.8 ± 3.2^b^
Duration of diabetes (years)	—	—	5.2 ± 5.8^a^	2.6 ± 4.4^b^	6.9 ± 5.7^c^
Systolic blood pressure (SBP; mmHg)	119.6 ± 13.5^a^	127.7 ± 12.9^bc^	123.1 ± 14.0^b^	129.3 ± 14.2^c^	126.7 ± 14.7^c^
Diastolic blood pressure (DBP; mmHg)	74.4 ± 9.7^a^	80.3 ± 11.0^bc^	76.2 ± 9.3^d^	80.7 ± 8.2^b^	77.3 ± 9.1^cd^
Central SBP (cSBP; mmHg)	121.4 ± 0.9^a^	133.1 ± 1.6^b^	127.9 ± 15.4^c^	142.4 ± 18.6^d^	135.0 ± 16.6^bd^
Central DBP (cDBP; mmHg)	76.9 ± 0.6^a^	83.7 ± 1.3^b^	77.2 ± 10.6^a^	82.9 ± 7.7^bc^	79.6 ± 10.1^c^
Pulse pressure (PP; mmHg)	44.5 ± 0.5^a^	49.4 ± 1.0^b^	50.7 ± 10.6^b^	59.5 ± 13.5^c^	55.4 ± 12.8^c^
Augmentation index (AI; %)	74.5 ± 12.1^a^	98.3 ± 9.3^b^	123.5 ± 182.0^c^	123.9 ± 261.7^c^	137.7 ± 180.9^c^
PWV, heart-carotid (hcPWV; m/s)	6.85 ± 0.10^a^	7.95 ± 0.25^bc^	7.73 ± 1.54^b^	8.19 ± 1.92^bc^	8.23 ± 1.89^c^
PWV, carotid-brachial (cbPWV; m/s)	4.42 ± 0.04^a^	4.75 ± 0.07^b^	4.73 ± 0.57^b^	4.90 ± 0.64^b^	4.76 ± 0.53^b^
PWV, heart-femoral (hfPWV; m/s)	8.15 ± 0.09^a^	8.84 ± 0.14^b^	9.53 ± 1.90^c ^	10.63 ± 2.04^d^	10.06 ± 2.17^d^

PWV: pulse wave velocity. Different letters within a variable are significantly different at *P* < 0.05.

**Table 3:** Frequencies of hypertension according to SNP genotypes.

			Prediabetes			Diabetes	
	Genotype	Normotensive	Hypertensive	*P* (corrected *P*)	Normotensive	Hypertensive	*P* (corrected *P*)
rs5326	GG/GA/AA	162/101/15	19/16/2	0.6726	256/133/16	178/116/24	0.0265
*(DRD1) *	(%)	(58.3/36.3/5.4)	(51.4/43.2/5.4)	(1)	(63.2/32.8/4)	(56/36.5/7.5)	(1)
rs1004467	TT/TC/CC	122/124/32	16/18/4	0.9949	160/215/34	157/130/29	0.0020
*(CYP17A1) *	(%)	(43.9/44.6/11.5)	(42.1/47.4/10.5)	(1)	(39.1/52.6/8.3)	(49.7/41.1/9.2)	(0.1040)
rs2960306	GG/GT/TT	221/59/4	31/9/0	0.4793	336/73/6	234/79/8	0.0066
(*GRK4) *	(%)	(77.8/20.8/1.4)	(77.5/22.5/0)	(1)	(81/17.6/1.4)	(72.9/24.6/2.5)	(0.3432)
rs11191548	TT/TC/CC	151/110/22	25/11/2	0.3199	216/182/16	197/102/22	0.0078
*(NT5C2) *	(%)	(53.4/38.9/7.8)	(65.8/28.9/5.3)	(1)	(52.2/44/3.9)	(61.4/31.8/6.9)	(0.4056)
rs1530440	CC/CT/TT	194/78/7	18/19/3	0.0015	287/116/9	222/89/5	0.8878
*(C10orf107) *	(%)	(69.5/28/2.5)	(45/47.5/7.5)	(0.0780)	(69.7/28.2/2.2)	(70.3/28.2/1.6)	(1)

**Table 4:** Associations of SNP genotypes and hemodynamic parameters.

Set	Parameters	rs number	Nearby gene (s)	Statistical methods	*P *	Corrected *P *
	SBP	rs6749447	*STK39 *	Linear regression	0.0100	0.5213
Prediabetes		rs2384550	*TBX3 *	Linear regression	0.0341	1
	DBP	rs699	*AGT *	Linear regression	0.0021	0.1079

	SBP	rs17249754	*ATP2B1 *	Linear regression	0.0480	1
	DBP	rs7926335	*PLEKHA7 *	Linear regression	0.0297	1
		rs381815	*PLEKHA7 *	Linear regression	0.0349	1
	cDBP	rs7926335	*PLEKHA7 *	Linear regression	0.0072	0.3754
		rs381815	*PLEKHA7 *	Linear regression	0.0096	0.4970
		rs1004467	*CYP17A1 *	Median regression	**0.0001**	**0.0060**
Diabetes without antihypertensive medication	AI	rs7926335	*PLEKHA7 *	Median regression	0.0316	1
		rs381815	*PLEKHA7 *	Median regression	0.0428	1
		rs16998073	*FGF5 *	Median regression	0.0105	0.5435
	hcPWV	rs10889553	*LEPR *	Linear regression	0.0472	1
		rs17097182	*LEPR *	Linear regression	0.0472	1
		rs4961	*ADD1 *	Linear regression	0.0043	0.2216
	hfPWV	rs1937506	*PCDH9 *	Linear regression	0.0032	0.1639
		rs1801058	*GRK4 *	Linear regression	0.0095	0.4932

	DBP	rs7961152	*BCAT1 *	Linear regression	0.0138	0.7169
	cSBP	rs2303934	*SLC4A2 *	Linear regression	0.0408	1
		rs2681492	*ATP2B1 *	Linear regression	0.0304	1
	cDBP	rs2681472	*ATP2B1 *	Linear regression	0.0257	1
		rs2303934	*SLC4A2 *	Linear regression	0.0249	1
		rs17249754	*ATP2B1 *	Linear regression	0.0292	1
		rs995322	*CSMD1 *	Linear regression	0.0355	1
	PP	rs10889553	*LEPR *	Linear regression	0.0242	1
		rs17097182	*LEPR *	Linear regression	0.0238	1
	AI	rs6495122	*CPLX3 *	Median regression	0.0281	1
		rs10889553	*LEPR *	Median regression	0.0485	1
Diabetes with antihypertensive medication		rs5443	*GNB3 *	Median regression	0.0485	1
	hcPWV	rs12946454	*ACBD4 *	Median regression	0.0202	1
		rs1530440	*C10orf107 *	Median regression	0.0198	1
		rs1799998	*CYP11B2 *	Median regression	0.0014	0.0725
		rs394112	*SLC8A1 *	Linear regression	0.0452	1
		rs10889553	*LEPR *	Linear regression	0.0260	1
	cbPWV	rs1378942	*CSK *	Linear regression	0.0210	1
		rs17097182	*LEPR *	Linear regression	0.0258	1
		rs17367504	*MTHFR *	Linear regression	0.0416	1
	hfPWV	rs2303934	*SLC4A2 *	Linear regression	0.0049	0.2527
		rs1937506	*PCDH9 *	Linear regression	0.0455	1

SBP: systolic blood pressure; DBP: diastolic blood pressure; cSBP: central systolic blood pressure; cDBP: central diastolic blood pressure; AI: augmentation index; PP: pulse pressure; hcPWV: heart-carotid pulse wave velocity; hfPWV: heart-femoral pulse wave velocity; cbPWV: carotid-brachial pulse wave velocity.
