# Supplementary material for: Genetic Variation in CYP17A1 Is Associated with Arterial Stiffness in Diabetic Subjects
Source: Exp Diabetes Res. 2012 Oct 23;2012:827172. doi: 10.1155/2012/827172 (PMC3485973; doi:10.1155/2012/827172)
Supplement: Supplementary file 1 — List of single nucleotide polymorphisms (SNP) investigated in the present study. [file 827172.f1.pdf]

Supplementary Table S1: List of single nucleotide polymorphisms (SNP) investigated in the present study.

**SNP ID**	**Nearby gene**
rs699	*AGT*
rs4532	*DRD1*
rs4961	*ADD1*
rs5186	*AGTR1*
rs5326	*DRD1*
rs5443	*GNB3*
rs265981	*DRD1*
rs381815	*PLEKHA7*
rs394112	*SLC8A1*
rs405884	*SLC8A1*
rs406222	*SLC8A1*
rs415695	*SLC8A1*
rs995322	*CSMD1*
rs1004467	*CYP17A1*
rs1024323	*GRK4*
rs1105297	*SLC2A1*
rs1378942	*CSK*
rs1530440	*C10orf107*
rs1799998	*CYP11B2*
rs1801058	*GRK4*
rs1937506	*PCDH9*
rs2303934	*SLC4A2*
rs2384550	*TBX3*
rs2398162	*LOC100132798*
rs2681472	*ATP2B1*
rs2681492	*ATP2B1*
rs2820037	*CHRM3*
rs2960306	*GRK4*
rs3754777	*STK39*
rs4149601	*NEDD4L*
rs6495122	*CPLX3*
rs6594013	*ATP2B4*
rs6749447	*STK39*
rs6997709	*KCNK9, COL22A1*
rs7926335	*PLEKHA7*
rs7961152	*BCAT1*
rs9815354	*ULK4*
rs10889553	*LEPR*
rs10954174	*LEP*
rs11014166	*CACNB2*
rs11110912	*MYBPC1*
rs11191548	*NT5C2*
rs11195417	*ADRA2A*
rs11195419	*ADRA2A*
rs12035521	*ATP2B4*
rs12945290	*ARSG*
rs12946454	*ACBD4*
rs16948048	*ZNF652*
rs16998073	*FGF5*
rs17097182	*LEPR*
rs17249754	*ATP2B1*
rs17367504	*MTHFR*
